# Optimizing Esthetics in Maxillary Anterior Periradicular Surgery: A Case Report on Vestibular Incision

**DOI:** 10.1155/crid/9688218

**Published:** 2026-10-21

**Authors:** Manish Rathi, Saravanan Sampoornam Pape Reddy, Delfin Lovelina Francis, Ujjwal Joshi

**Affiliations:** ^1^ Department of Periodontology Army Dental Centre (Research & Referral) New Delhi India; ^2^ Saveetha Dental College & Hospitals Saveetha University SIMATS Chennai India saveetha.com; ^3^ Department of Oral Medicine and Radiology Kathmandu Medical College Teaching Hospital Kathmandu Nepal kmc.edu.np

## Abstract

**Background:**

Persistent periapical infections may necessitate surgical intervention when nonsurgical endodontic treatment fails. Conventional surgical approaches in the maxillary anterior region often lead to visible scarring and prolonged healing, compromising esthetic outcomes. The vestibular incision subperiosteal tunnel access (VISTA) technique offers a minimally invasive alternative that preserves soft tissue integrity and reduces scarring.

**Case Report:**

A 32‐year‐old male presented with symptomatic apical periodontitis of the maxillary left central incisor; universal Tooth #9 following failed nonsurgical retreatment. Periapical surgery was performed using the VISTA approach via a vestibular incision beneath the maxillary labial frenum to access the lesion with minimal tissue trauma. After debridement and apicoectomy, the defect was filled with a demineralized xenograft and covered with L‐PRF membrane. The flap was sutured for optimal healing. Postoperative follow‐up at 12 months demonstrated complete radiographic resolution of the periapical lesion, excellent soft tissue healing without visible scarring, and patient‐reported minimal discomfort and anxiety.

**Conclusion:**

This case illustrates a potential application of the VISTA approach in anterior periradicular surgery, a well‐established method in periodontal plastic surgery. The integration of VISTA with regenerative materials, such as xenografts and platelet‐rich fibrin (PRF), may enhance bone healing and esthetic outcomes, thereby generating hypotheses for future research.

**One-Sentence Summary**

This case report describes the vestibular incision subperiosteal tunnel access (VISTA) incision method combined with platelet‐rich fibrin (PRF) and xenografts, which facilitates minimally invasive, cosmetic, and effective treatment of recurrent periapical disease, resulting in successful bone regeneration.

## 1. Introduction

Periapical pathology is a common endodontic condition characterized by inflammation and progressive destruction of periapical tissues, often due to microbial infection of the root canal system. It appears as pain, swelling, tenderness to percussion, and radiographically, as a radiolucent lesion with osteosclerosis [1]. Surgical intervention is indicated when traditional nonsurgical endodontic treatment is unsuccessful in the management of periapical disease, facilitating the removal of infection and allowing for healing and preservation of tissue integrity [2]. Periapical surgery (curettage, apicoectomy, and retrograde filling) is a well‐meaning procedure for failed root canal therapy. Nevertheless, ideal access and optimal esthetic and functional results are not always easy to achieve, especially in the maxillary anterior dentition. Conventional incisions can cause postoperative scarring and prolonged healing, compromising the esthetics of the surgery.

VISTA is a minimally invasive operative approach that allows access to the operative site while minimizing mucosal trauma and maximally preserving esthetics. By making the incision deliberately, such as in proximity to the maxillary labial frenum, the periapical area can be accessed, minimizing the scarring and aiding recovery [3]. The versatility of VISTA enhances its utility across multiple contexts, including its role within surgically facilitated orthodontic therapy (SFOT) protocols. Thus, it facilitates orthodontic interventions by allowing soft tissue manipulation and graft placement with reduced morbidity [4]. A recent systematic review and meta‐analysis on VISTA application with other biomaterials including PRF showed significant benefits in terms of increasing keratinized tissue width [5].

Therefore, the VISTA incision, which is primarily introduced for periodontal plastic surgeries, can be explored for periapical access as it utilizes the principle of minimal trauma, esthetic preservation, and minimizing the postoperative discomfort.

This report presents a 32‐year‐old patient with recurrent periapical disease associated with a left maxillary central incisor (Tooth #9). After unsuccessful nonsurgical endodontic therapy, periapical surgery was performed with the VISTA flap and placement of a xenograft and PRF, aimed at regenerating the osseous defect.

## 2. Material and Methods

This case report has been written according to the Preferred Reporting Items for Case Reports in Endodontics (PRICE) 2020 guidelines [6], with duly filled PRICE checklist and flowchart (Table 1 and Figure 1), and was conducted in accordance with the Declaration of Helsinki and approved by the institutional review board where applicable. A 32‐year‐old male presented with ongoing pain related to the maxillary left central incisor, universal Tooth #9. His past medical history was noncontributory and was in ASA physical status class I. His past dental history revealed that he had undergone endodontic treatment for Tooth #9 2 years earlier. Clinical examination revealed tenderness to percussion and no detectable mobility (Figure 2). Radiographic examination revealed a confined periapical radiolucency with inadequate lateral and apical sealing of the obturating material and extrusion of the root canal sealer beyond the apex, indicating recurrent periapical disease. Nonsurgical endodontic retreatment attempted 3 months prior had failed. The patient′s general intraoral findings, including periodontal condition, were healthy. To confirm the extent of the lesion, the patient was advised to undergo cone‐beam computed tomography (CBCT), which the patient declined due to financial and radiation concerns. The absence of three‐dimensional imaging (CBCT) limits comprehensive characterization of the periapical lesion and surgical planning precision. Without CBCT, the lesion′s spatial extent and relationship to adjacent vital structures cannot be fully assessed, potentially affecting precise surgical access and planned debridement.

**Table 1:** PRICE guidelines. Checklist of items to be included when reporting case reports in endodontics.

Section/topic	Item number	Checklist item	Reported on page number
Title	1a	The words “case report(s)” must be included in the title.	01
	1b	The area of interest (e.g., anatomy, disease, and treatment) must be included briefly in the title.	01

Keywords	2a	At least two relevant keywords, preferably MeSH terms, related to the content of the case report must be included.	01

Abstract	3a	The introduction must contain information on how the report is novel and contributes to the literature, clinical practice, and/or fills a gap(s) in knowledge.	02
	3b	The body must describe the main clinical findings, including symptoms and signs, if present.	01
	3c	The body must describe the main radiographic/histological/laboratory/diagnostic findings.	01
	3d	The body must describe the main outcomes of treatment, if active treatment has been provided.	01
	3e	The conclusion(s) must contain the main “take‐away” lesson(s), sometimes referred to as key learning point(s).	01‐02

Introduction	4a	A background summary of the case(s) with relevant information must be provided.	02

Informed consent	5a	A clear statement that informed, valid consent was obtained from the patient(s) must be provided.	04

Case report information	6a	The age of the patient(s) must be provided.	03
	6b	The gender of the patient(s) must be provided.	03
	6c	The ethnicity of the patient(s) must be provided, if relevant.	Not relevant
	6d	The main concern, chief complaint or symptoms of the patient(s), if any, must be provided.	03
	6e	The medical history of the patient(s) must be provided, if relevant.	03
	6f	The dental history of the patient(s) must be provided, if relevant.	03
	6g	The family history of the patient if associated with the primary complaint must be provided, if relevant.	Not relevant
	6h	The psychosocial history of the patient if associated with the primary complaint must be provided, if relevant.	Not relevant
	6i	Genetic information, including details of relevant comorbidities and past interventions and their outcomes must be provided when possible, if relevant.	Not relevant
	6j	Extraoral findings must be provided, if relevant.	Not relevant
	6k	General intraoral findings must be provided when relevant, for example, carious lesions, restorations, periodontal condition, and soft tissues.	04
	6l	Important/relevant dates and times (in the text, or a table or figure) must be provided in chronological order.	16
	6m	The diagnostic methods and the results for the specific tooth/teeth (e.g., pulp sensibility test, tenderness, mobility, periodontal probing depths, laboratory investigations, imaging techniques, or other special tests) must be provided.	04
	6n	The diagnostic challenges, if any, must be provided.	04
	6o	The diagnostic reasoning including other possible diagnoses that were considered must be provided.	04
	6p	The active treatment(s) or intervention(s) performed, if any, must be provided.	04
	6q	Any modifications to the proposed treatment(s) or intervention(s), if necessary, must be provided.	04
	6r	The assessment method(s) used to determine the clinician‐assessed and patient‐assessed treatment outcomes and their results must be provided.	04
	6s	Adverse and unanticipated events or consequences, if any, must be provided.	NIL

Discussion	7a	The specific treatment(s) and intervention(s) (if any) must be discussed with reference to the relevant literature.	05
	7b	The strengths of the case report and its importance must be discussed with reference to the relevant literature.	06
	7c	The limitations of the case report must be discussed.	07
	7d	The rationale for the conclusion(s) must be discussed.	07

Patient perspective	8a	Feedback from the patient on the treatment and the care they received should be provided, if relevant.	04

Conclusion	9a	Explicit conclusion(s), that is, the main “take‐away” lessons must be provided.	09
	9b	Implications for clinical practice or future research must be provided.	09

Funding details	10a	Sources of funding and other support (such as supply of instruments, and equipment) as well as the role of funders must be acknowledged and described.	09

Conflicts of interest	11a	An explicit statement on conflicts of interest must be provided.	09

Quality of images	12a	Details of the equipment, software, and settings used to acquire the image(s) must be described in the text or legend.	16
	12b	The reason why the image(s) was acquired and the rationale for its inclusion in the manuscript must be provided in the text.	16
	12c	The circumstances (conditions) under which the image(s) were viewed and evaluated by the authors must be provided in the text.	16
	12d	The resolution and any magnification of the image(s) or any modifications/enhancements (e.g., adjustments for brightness, color balance, or magnification, image smoothing, and staining) that were carried out must be described in the text or legend.	16
	12e	Patient(s) identifiers (names and patient numbers) must be removed to ensure they are anonymised.	13‐16
	12f	An interpretation of the findings (meaning and implications) from the image (s) must be provided in the text.	4
	12g	The legend associated with each image must describe clearly what the subject is and what specific feature(s) it illustrates. Legends associated with images of patients must describe the age, gender, and ethnicity of the person, if relevant.	16
	12h	Markers/labels must be used to identify the key information in the image(s) and be defined in the legend or as a footnote.	16
	12i	The legend of each image must include an explanation whether it is pretreatment, intratreatment, or posttreatment and, if relevant, how images over time were standardized.	16

**Figure 1:**
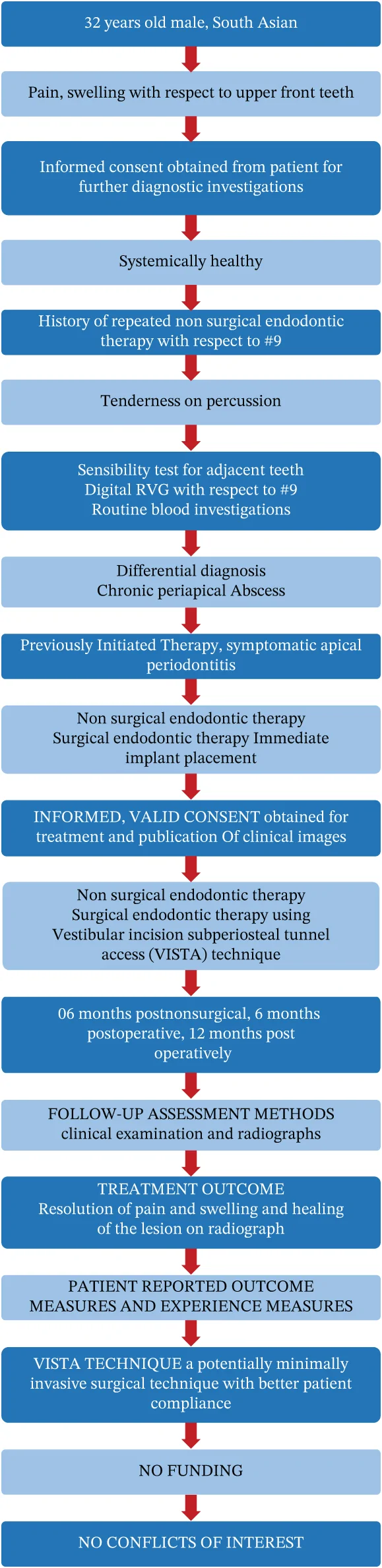
PRICE flowchart. ∗From: Nagendrababu V, Chong BS, McCabe P, Shah PK, Priya E, Jayaraman J, Pulikkotil SJ, Setzer FC, Sunde PT, Dummer PMH (2020) PRICE 2020 Guidelines for Reporting Case Reports in Endodontics: A Consensus‐Based Development. *International Endodontic Journal* doi:10.1111/iej.13285. For further details visit http://pride-endodonticguidelines.org/price/.

**Figure 2:**
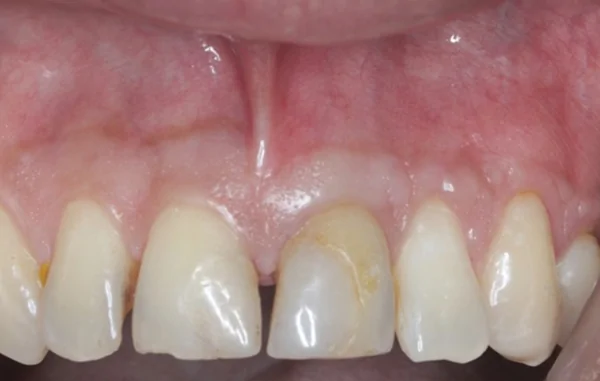
Preoperative clinical situation related to Tooth #9 (September 13, 2023).

After correlating the clinical and radiographic features seen on IOPA, a diagnosis of “Previously treated Chronic Apical Abscess” was made. Initially, nonsurgical endodontic retreatment was performed, and the patient was kept under observation for 6 months. However, due to persistent periradicular pathosis and tenderness, the patient reported discomfort in the periapical region with respect to Tooth #9 on palpation in the vestibular region as well as percussion, the decision to perform the surgical endodontics was made. Surgical endodontics was preferred as the case had been retreated nonsurgically multiple times previously, and it was clinically impossible to adequately debride the apical 3rd of the root along with the accessory canals. Radiography revealed no significant healing after retreatment and persistent periapical radiolucency (Figure 3b,c). Therefore, periapical surgery was performed to address the apical pathology by removing the lesion. The patient provided written informed consent for the treatment and publication of clinical photographs. A VISTA incision, as previously described by Zadeh [3] for anterior root coverage procedures, was selected to access the surgical site for treating the periapical pathology in Tooth #9. The incision was strategically placed underneath the maxillary labial frenum to minimize trauma, prevent scar formation, and achieve direct access to the periapical region of Tooth #9 (Figure 4). The incision extended from the mucogingival junction through the frenum to the alveolar mucosa and up to the vestibular fornix. The mucoperiosteal flap was meticulously raised to reveal the apical region of Tooth #9, thereby facilitating comprehensive debridement and curettage (Figure 5). After debridement (Figure 6), upon clinical observation of healthy bone, apicoectomy was performed to eliminate the accessory canals in the apical third region of the root, and retrograde sealing of the root canal was performed using mineral trioxide aggregate (ProRoot MTA; Dentsply Sirona). A periapical regeneration technique was executed using a combination of demineralized xenograft^#^(DMBM, 250 micron particle size; bovine derived) followed by the application of L‐PRF. L‐PRF was prepared after venous blood (10 mL) was collected and placed in a centrifuge (REMI R‐4C Model R‐43; fixed‐angle rotor). To achieve an effective g‐force of 400 × g for L‐PRF preparation, centrifugation was carried out at 2700 rpm for 12 min in sterile glass test tubes, and the resultant PRF clot was removed (Figures 7 and 8). The flap was secured and sutured using 5/0 polyglactin sutures to achieve hemostasis (Figure 9). Postoperative instructions were given to patients along with analgesic (ibuprofen 400 mg thrice daily for 3 days).

**Figure 3:**
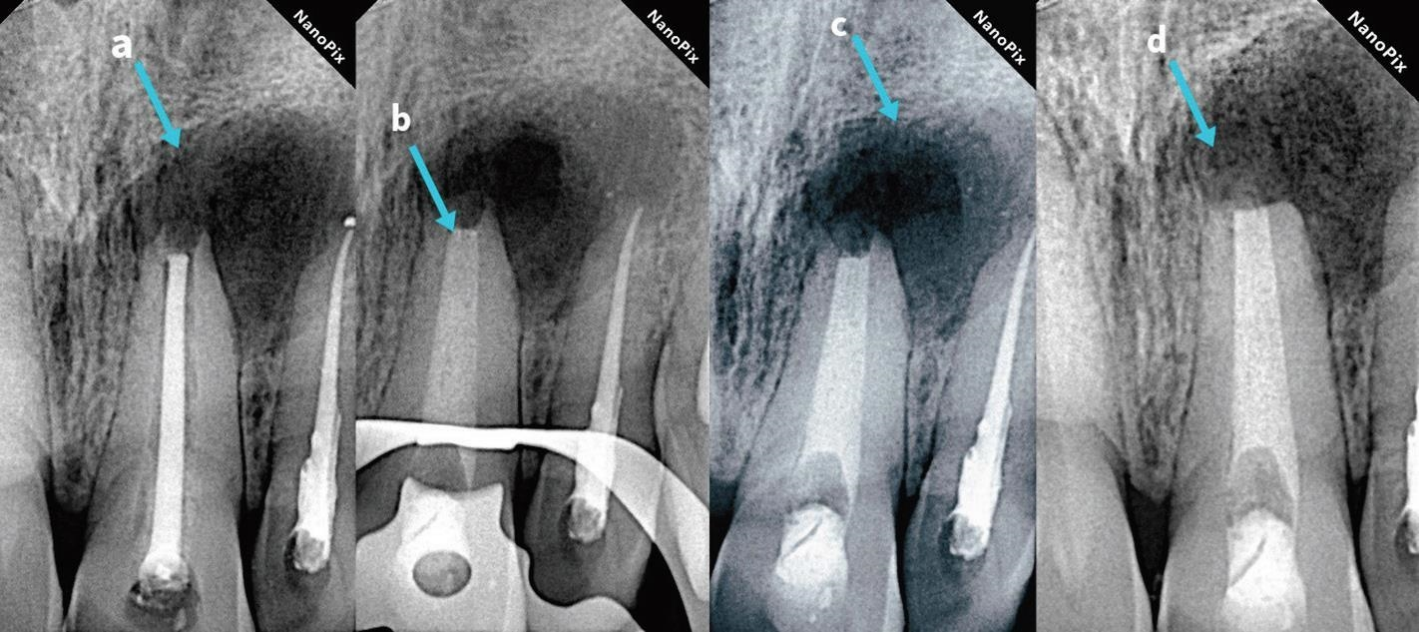
(a) Preoperative intraoral periapical radiograph showing extent of initial lesion, (b) RVG revealing completion of endodontic re‐treatment and MTA filling, (c) a 6‐month follow‐up showing insufficient periradicular healing, and (d) postoperative intraoral periapical radiograph (12‐month follow‐up) showing root end resection as well substantial healing of radiolucent lesion.

**Figure 4:**
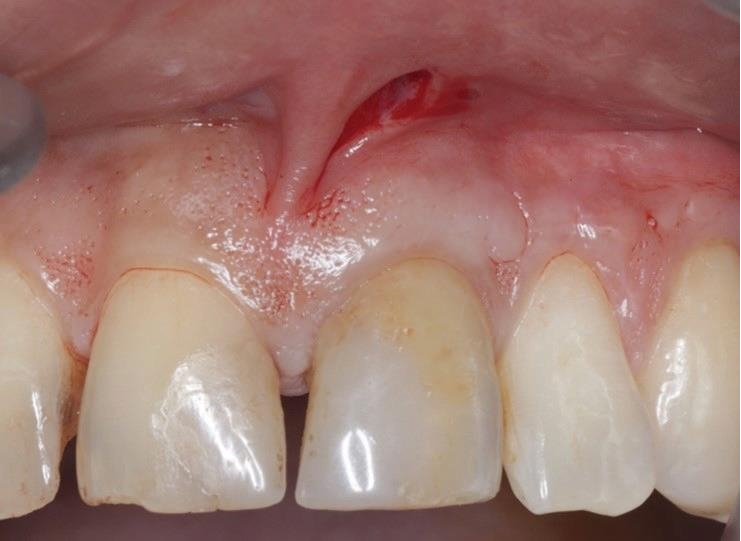
Midline incision in the maxillary labial frenum related to Tooth #9 (September 21, 2023).

**Figure 5:**
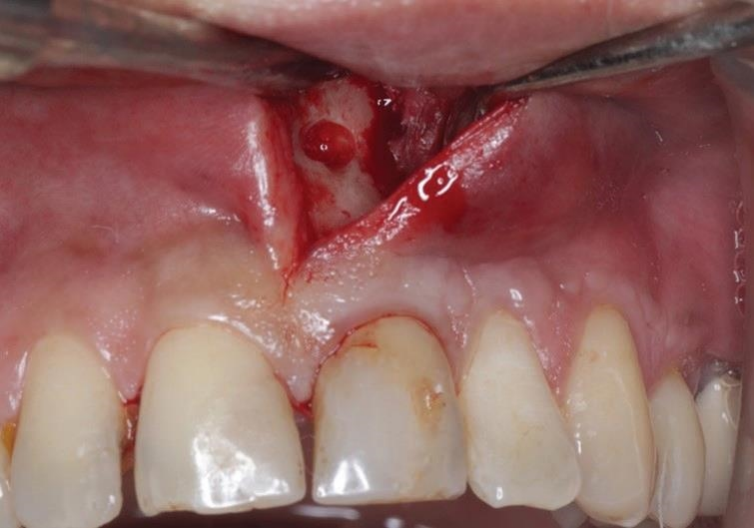
Full thickness subperiosteal tunnel preparation related to Tooth #9.

**Figure 6:**
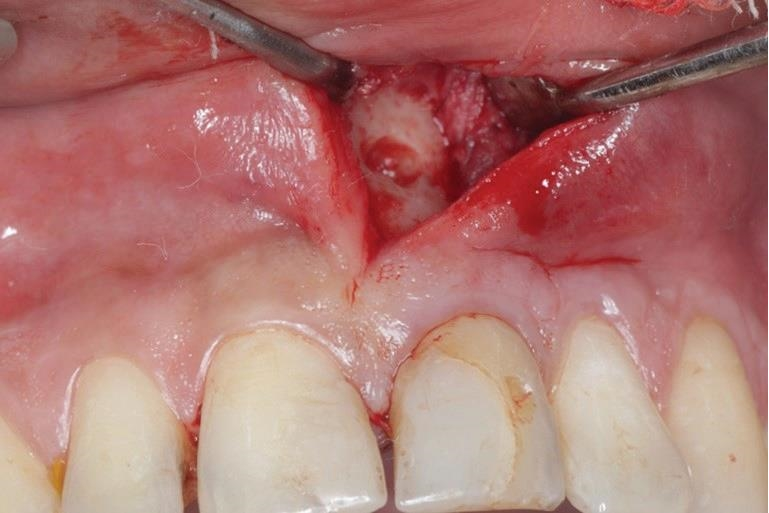
Completion of debridement related to Tooth #9.

**Figure 7:**
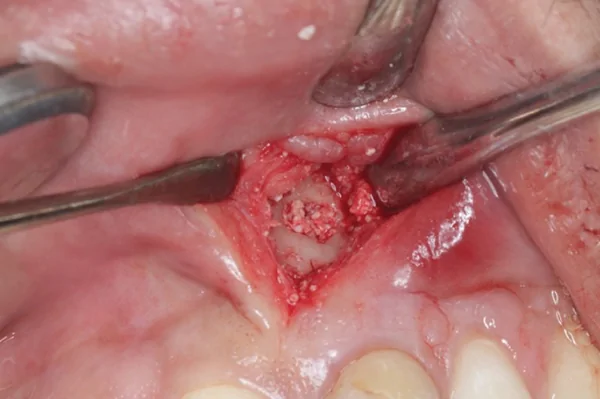
Placement of xenogeneic bone replacement graft.

**Figure 8:**
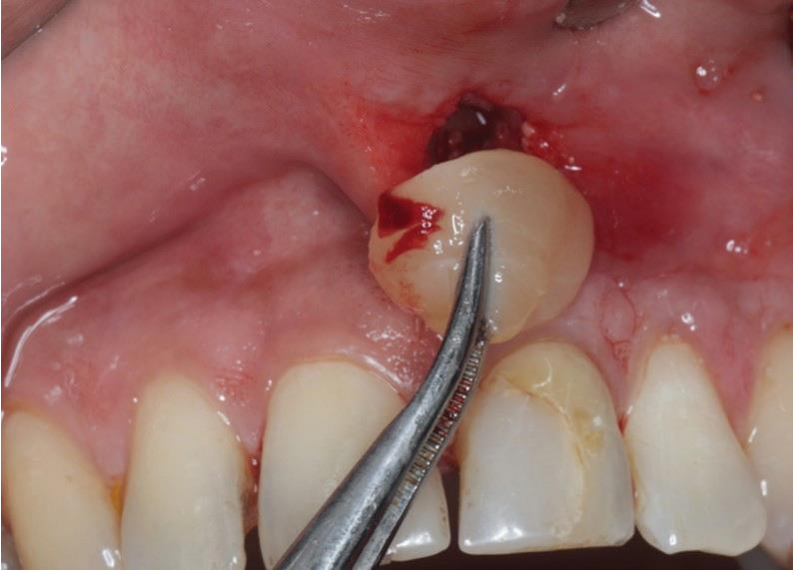
Placement of leukocyte‐ and platelet‐rich fibrin.

**Figure 9:**
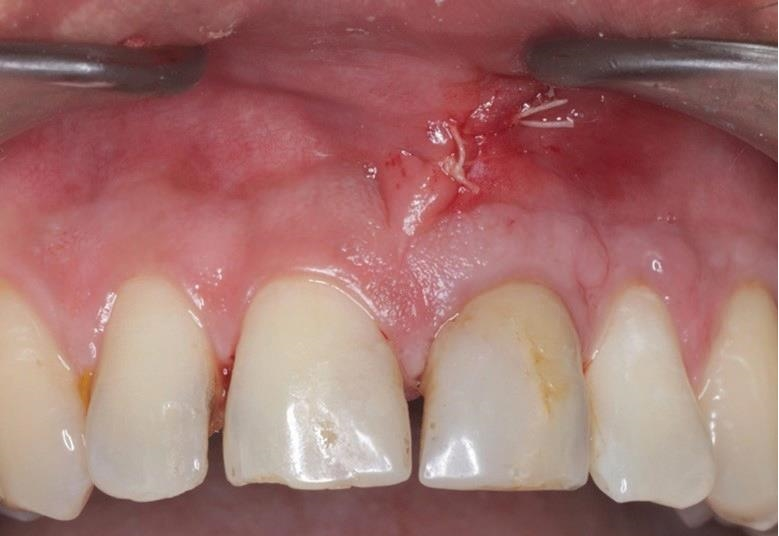
Flap closure using interrupted sutures in the frenal region.

## 3. Results

The patient experienced minimal postoperative discomfort with no complications. The 48‐h postoperative examination showed satisfactory early wound healing without suppuration or exudation (Figure 10). Follow‐up examinations showed scar‐free healing of the surgical site with excellent tissue healing and esthetic outcomes (Figure 11). On radiography, the preoperative image showed periapical radiolucency with inadequately obturated canals (Figure 3a), which was then managed by nonsurgical retreatment (Figure 3b) and subsequently reviewed at 6 months (Figure 3c). Postoperative radiographic healing showed resolution of the periapical radiolucency, which was confirmed 12 months postoperatively (Figure 3d). Patient‐reported outcomes included less postoperative pain and discomfort. The patient subjectively reported minimal postoperative discomfort. Postoperative healing was measured in terms of Mucosal Scarring Index (MSI). The MSI is an index based on width, height/contour, color, suture marks, and overall appearance and each parameter is graded with a 0–1–2 score, yielding a MSI score ranging from 0 (*no scar*) to 10 (*most extreme scar*) [7]. MSI was recorded postoperatively at 48 h, 1 week, and 1 month. The score recorded at 48 h was 06/10, suggestive of acceptable healing, which subsequently improved to 0/10 at 1 month postoperatively suggestive of excellent healing outcomes (Table 2). Therefore, postoperative healing and the minimal discomfort that the patient experienced may be related to use of VISTA incision as a minimally invasive approach. Regenerative outcomes may be related to use of bone grafts along with platelet concentrates. However, these outcomes, despite being positive, are purely subjective and are subject to exploration.

**Figure 10:**
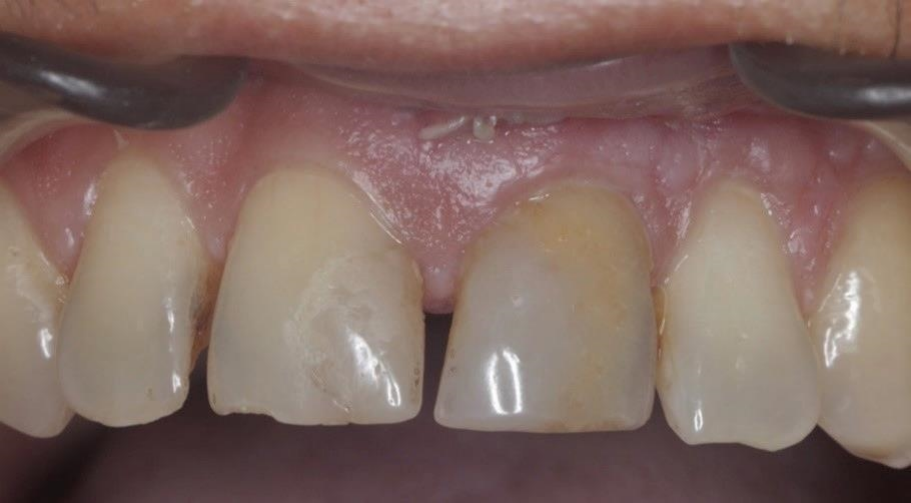
Postoperative clinical situation at 48 h after surgery (September 23, 2023).

**Figure 11:**
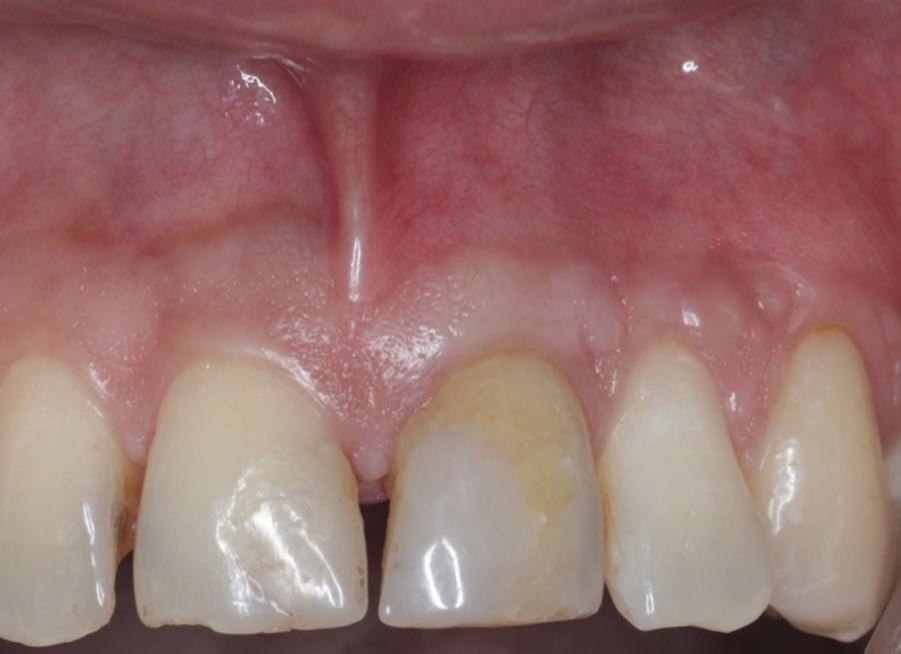
Postoperative clinical situation 12 months after surgery (October 14, 2024).

**Table 2:** Mucosal Scarring Index.

S. No.	Parameters	Score 48‐h post‐op	Score 1‐week post‐op	Score 1‐month post‐op
1.	Width	1	0	0
2.	Height/contour	1	0	0
3.	Color	2	0	0
4.	Suture marks	1	1	0
5.	Overall appearance	1	0	0
	Total	06	01	0

## 4. Discussion

Traditionally, incision techniques in periapical surgery involve sulcular, paramarginal, or vertical releasing incisions, which can result in visible scarring and compromise the esthetic outcome, especially in the anterior maxillary region. This case highlights the VISTA incision technique as a potentially minimally invasive approach to access the periapical region that overcomes the limitations of conventional incisions. The VISTA approach innovatively places incisions in inconspicuous regions, such as the maxillary labial frenum, thus minimizing trauma and preserving the vascular integrity. Therefore, Zadeh et al. [4] also support this decision when they recommend the preservation of soft tissue architecture in regenerative approaches, as this strategy helps in achieving predictable healing outcomes. The techniques take advantage of minimizing ischemia and necrosis, and as a result, faster healing and soft tissue regeneration occur due to no vertical incisions [8]. The tunnel created subperiosteal tunnel allows for direct access to the lesion site with minimum tissue reflection, which is critical to maintaining tissue integrity. By maintaining a considerable distance between the surgical site and esthetic zone, the procedure effectively minimizes the risk of scarring, thereby significantly enhancing the postoperative appearance of patients. The subperiosteal tunnel created follows a VISTA incision for direct access to the periapical lesion allowing enough access for adequate debridement and apicoectomy if needed [9].

This approach also allows concomitant regenerative therapies, such as xenografts and PRF, to be placed without additional incisions. PRF is a second‐generation growth factor‐rich platelet concentrate containing the following growth factors: transforming growth factor‐*β*1 (TGF‐*β*1), platelet‐derived growth factor (PDGF), and vascular endothelial growth factor (VEGF), which enhance fibroblast proliferation, angiogenesis, and extracellular matrix formation [10].

PRF enhances healing in endodontic surgery by releasing growth factors that support tissue regeneration and bone formation [11]. Its use in periapical procedures has demonstrated superior results, including faster healing, regeneration, reduced postoperative discomfort, and minimal scarring [12–14].

Deproteinized bovine bone mineral is an example of a xenograft extensively used in periapical surgery to fill osseous defects, which acts as a scaffold as it is an osteoconductive material [15]. Additionally, xenografts maintain space and prevent soft tissue collapse into the defect site, promoting optimal conditions for bone repair [16]. The 12‐month periapical radiograph showed radiographic findings consistent with substantial healing of the lesion. Radiographic bone healing after periapical surgery is typically assessed over 6–12 months. However, complete healing may occur over a longer time scale, and substantial bone formation is generally observed within this timespan. Follow‐up radiographs are performed on a routine basis to evaluate healing and to verify the resolution of the periapical lesions [17]. Follow‐up post‐12 months may indicate unaddressed pathophysiology and necessitate re‐evaluation of case and intervention. Even though 6–12‐month intervals are the common recommendations, individual variations emphasize the importance of patient‐specific follow‐up regimen aimed at optimizing treatment outcomes in each patient [18].

The current findings align with those of previous research, indicating that the incorporation of PRF into xenografts can enhance bone healing and regeneration. Dohan Ehrenfest et al. [19] demonstrated significant improvements in bone regeneration when PRF was utilized as an adjuvant in endodontic procedures, suggesting its efficacy in promoting healing. The VISTA approach minimizes trauma and esthetic concerns compared with traditional incision techniques, such as sulcular or vertical releasing flaps. von Arx et al. [20] noted that conventional flaps, while providing access, often result in postsurgical scarring and prolonged healing times. In this case, the VISTA approach appeared to provide favorable soft tissue healing; however, comparative studies are needed to determine whether it offers advantages over conventional flap designs. VISTA minimizes trauma to the gingival margin and papilla by avoiding sulcular incisions, reducing postoperative gingival recession and improving esthetic outcomes.

Patient‐reported outcomes such as pain, swelling, and bruising tend to be lower after minimally invasive procedures compared with conventional open flaps, with shorter surgery duration contributing to improved postoperative comfort [21]. Additionally, minimally invasive approaches like VISTA may reduce operative time and improve efficiency, similar to advantages observed in other personalized surgical methods employing precision techniques [22].

VISTA‐assisted periradicular surgery is especially indicated in cases where preservation of natural dentition is prioritized, especially in young patients having large/persistent periradicular defects after nonsurgical endodontic treatment. It is indicated when teeth exhibit internal root resorption or large periapical lesions posing a poor prognosis for nonsurgical retreatment but where extraction is less desirable due to age or esthetic concerns.

The VISTA technique has a few limitations as it requires precision, as lack of expertise may lead to tissue perforation or insufficient access to the lesion. Although effective for anterior teeth, the incision may be challenging to apply in posterior areas where access and visualization are restricted [23]. VISTA‐assisted periapical surgery is likely most appropriate for anterior maxillary teeth in patients who have high esthetic requirements and suitable anatomy, and it should not be assumed to be widely applicable to posterior teeth or complex lesions.

The outcomes of this case align with those of previous studies advocating minimally invasive techniques combined with regenerative materials for periapical surgery. A randomized controlled experiment by Pecora et al. [24] indicated that minimally invasive incisions combined with autologous biomaterials led to enhanced bone healing and fewer postoperative problems relative to conventional methods. Similarly, Kim et al. [25] in 2018 compared retreatment using endodontic microsurgery to primary endodontic surgery and found comparable outcomes in both groups. Moreover, the scar‐free healing observed in this case underscores the esthetic advantages of the VISTA approach. The radiographic appearance consistent with healed lesion in this case aligns with findings from von Arx et al. [26], who documented a substantial decrease in lesion size after regenerative periapical surgery. Nonetheless, two‐dimensional x‐rays are not dependable for accurately measuring actual bone fill or differentiating between scar tissue and newly formed bone, especially in the absence of CBCT or histological verification. The rationale for adapting the VISTA incision, a periodontal plastic surgery technique, to endodontic microsurgery is clinically important, addressing specific surgical challenges and has the potential to improve treatment outcome. However, these clinical benefits are attributable to multiple simultaneous interventions, including VISTA access, apicoectomy, retrograde MTA filling, xenograft placement, and L‐PRF.

This case study is subject to several other limitations, including its single‐case design, the absence of CBCT, the lack of a comparator flap design, lack of quantitative assessment of patient reported outcomes and esthetic satisfaction, and the unavailability of the histopathological image for diagnostic confirmation, as well as a short‐to‐intermediate follow‐up period for assessing definitive periapical healing.

The VISTA incision approach in periapical surgery opens avenues for further research and clinical applications. Future studies should focus on large‐scale randomized clinical trials comparing VISTA incision with traditional incision techniques to validate its superiority.

## 5. Conclusion

The VISTA approach allows esthetic and minimally invasive access for anterior periradicular surgery, promoting scar‐free healing and optimal tissue preservation when combined with regenerative materials. This case demonstrates the minimally invasive nature of VISTA incision, preserving soft tissue integrity and reducing postoperative discomfort.

## Author Contributions

All listed authors have made a substantial contribution to the manuscript. M.R. performed all the surgical procedures and follow‐ups, along with initial manuscript drafting. S.S.P.R. was involved in conceptualization, completed the case analysis, and prepared the final manuscript. D.L.F. revised the manuscript critically for important intellectual content and proofreading of the manuscript. U.J. was involved in the conceptualization revised the manuscript critically for important intellectual content.

## Funding

No funding was received for this manuscript.

## Disclosure

U.J. gave the final approval of the version to be published.

## Ethics Statement

Consent in writing was obtained from the patient for their medical treatment, the associated documentation and clinical images, as well as for the publication of this case report.

## Conflicts of Interest

The authors declare no conflicts of interest.

## Endnotes

^#^Osseograft, Advanced Biotech Private Limited, Chennai, India

## Data Availability

The data that support the findings of this study are available from the corresponding author upon reasonable request.
